# Estrogen Induces Metastatic Potential of Papillary Thyroid Cancer Cells through Estrogen Receptor ****α**** and ****β****

**DOI:** 10.1155/2013/941568

**Published:** 2013-10-10

**Authors:** Wenwu Dong, Hao Zhang, Jing Li, Haixia Guan, Liang He, Zhihong Wang, Zhongyan Shan, Weiping Teng

**Affiliations:** ^1^Department of General Surgery, The First Affiliated Hospital of China Medical University, No. 155 Nanjing Bei Street, Heping District, Shenyang, Liaoning 110001, China; ^2^Department of Endocrinology and Metabolism, Institute of Endocrinology, Liaoning Provincial Key Laboratory of Endocrine Diseases, The First Affiliated Hospital of China Medical University, No. 155 Nanjing Bei Street, Heping District, Shenyang, Liaoning 110001, China

## Abstract

Estradiol (E2) promotes metastatic propensity. However, the detailed mechanism remains largely unknown. E-cadherin, vimentin, and MMP-9 play a dominant role in the metastatic process. We aimed to investigate the effects of E2 on metastatic potential of PTC cell line BCPAP and on E-cadherin, vimentin, and MMP-9 protein expression. PTC cell line BCPAP was evaluated for the presence of estrogen receptor (ER) by western blot analysis. The effects of E2, PPT (a potent ER**α**-selective agonist), and DPN (a potent ER**β**-selective agonist) on modulation of metastatic phenotype were determined by using *in vitro* scratch wound assay and invasion assay. In addition, the effects on E-cadherin, vimentin, and matrix metalloproteinase-9 (MMP-9) protein expression were evaluated by Western blot analysis. We found that BCPAP cells expressed ER**α** and ER**β**. E2 and PPT enhanced, but DPN inhibited, the migration and invasion of BCPAP cells in an *in vitro* experimental model system that is modulated by E-cadherin, vimentin, and MMP-9. These findings indicate that E2 induces the metastatic potential of BCPAP cells through ER**α** and ER**β**. The two ER subtypes play differential roles in modulation of BCPAP cell metastasis and the related molecule expressions including E-cadherin, vimentin, and MMP-9.

## 1. Introduction

Thyroid cancer is the most common cancer of the endocrine system and the sixth most common cancer in women in the United States [1]. Thyroid cancers are classified into 3 main histologic types as differentiated (including papillary, follicular, and Hürthle), medullary, and anaplastic (aggressive undifferentiated tumor), with papillary thyroid cancer (PTC) accounting for 80% of all cases. Differentiated thyroid cancer (DTC) has a faveorable long-term prognosis, with a 10-year relative survival rate of more than 90%. However, up to 10% of DTC invades through the outer border of the gland and grows directly into surrounding tissues, increasing both morbidity and mortality. Recurrence rates are 2 times higher with locally invasive tumors, and as many as 33% of patients with such tumors die of cancer within a decade [2, 3]. DTC can metastasize to the lymph nodes of the neck in 20–90% of the patients [4]. Distant metastasis, which occurs in 5–23% of patients with known DTC, is the most common cause of death in these patients [5]. Therefore, great efforts should be made to improve the prognosis of these patients with aggressive tumor.

Clinical and epidemiological studies have shown that the female predominance in thyroid cancer is the greatest during reproductive age and the incidence decreases after menopause [6, 7]. The use of oral contraceptives appears to result in a moderately increased risk of developing thyroid cancer and an elevated risk was also reported in women who used estrogens for gynecological problems, but not for low-dose estrogen replacement therapy in postmenopausal women. There are also experimental lines of evidence that E2 could affect tumor progression by increasing cell proliferation as well as promoting invasion or cell mobility in thyroid cancer [8–10]. However, the underlying molecular mechanism of E2 on promoting PTC progression is still unclear.

The regulation of gene expression by E2 is a multifactorial process, involving both genomic and nongenomic actions that converge at certain response elements located in the promoters of target genes [11]. In the genomic pathway, estrogens bind to two estrogen receptor (ER) subtypes, ER*α* and ER*β*, inducing an activating conformational change within the ER, promoting dimerization and high-affinity binding to specific estrogen response elements (EREs) located within the regulatory regions of target genes. ER*α* and ER*β* show significant overall sequence homology, but the different sizes of the binding pockets and sequences of their activation function domains indicate that ER*α* and ER*β* should have different specificities for ligands and biological responses [12]. Thus, ER*α* and ER*β* selective agonists are promising new approach for treating specific conditions associated with endocrine-related disease. Besides that, estrogens are also able to exert nongenomic events mediated by a novel transmembrane ER G protein-coupled receptor 30 (GPR30) [13].

The metastatic process, that is, the dissemination of cancer cells throughout the body to seed secondary tumors at distant sites, requires cancer cells to leave the primary tumor and to acquire migratory and invasive capabilities. Many different processes are involved in tumor cell invasion and metastasis such as an epithelial to mesenchymal transition (EMT), adhesion molecules downregulation, and matrix metalloproteinases (MMPs) upregulation in cancer cells. Loss of epithelial protein marker E-cadherin, the concurrent upregulation of mesenchymal protein markers vimentin, and the upregulation of MMP-9 play a dominant role in the metastatic process and could be regulated by E2 in various cancers, including breast, ovarian, colon, and lung cancer [14–17]. However, whether and how the effects of E2 on migration and invasion of thyroid cancer may be involved in the regulation of E-cadherin, vimentin, and MMP-9 is poorly understood.

In this study, we explore the effects of E2, PPT, and DPN on metastatic potential of PTC cell line BCPAP and evaluate the roles of ER*α* and ER*β* in metastasis of BCPAP cells. We further determine the metastasis-related proteins by which E2 affects BCPAP cell metastasis.

## 2. Materials and Methods

### 2.1. Cell Culture

The human PTC cell line BCPAP was purchased from DSMZ (Braunschh, Germany). The human breast cancer cell line MCF-7 was obtained from the Cell Bank of the Chinese Academy of Sciences (Shanghai, China). The cells were maintained in RPMI1640 medium supplemented with 10% fetal bovine serum, 100 U/mL penicillin, and 100 *μ*g/mL streptomycin in a humidified atmosphere of 5% CO_2_ at 37°C.

### 2.2. Treatments

Cells were stripped from endogenous steroids by changing the medium to phenol red-free RPMI1640 containing 10% charcoal-dextran stripped fetal bovine serum (FBS) (Biowest, Nuaillé, France) 48 h before treatment. Cells were then incubated in fresh medium with vehicle (DMSO) alone, 10^−8 ^M E2 (Sigma, St. Louis, MO), 10^−6 ^M PPT (an ER*α*-selective agonist) (Tocris, Ballwin, MO), or 10^−6 ^M DPN (an ER*β*-selective agonist) (Tocris, Ballwin, MO) for 24 h. Concentrations of E2, PPT, and DPN are used according to the report by Zeng et al. Control cultures received the same volume of DMSO.

### 2.3. Western Blot Analysis

Total protein was extracted using Total Protein Extraction Kit (KeyGEN, Nanjing, China) according to the manufacturer's instructions. Protein concentration was determined using a Bradford assay with bovine serum albumin as a standard. Protein was resolved with 10% SDS-PAGE and then electrophoretically transferred to polyvinylidene difluoride (PVDF) membranes (Millipore, CA, USA). The membranes were blocked with 5% nonfat dry milk at room temperature for 1 h and incubated at 4°C overnight with antibodies directed against ER*α* (Abcam, USA, 1 : 500), ER*β* (Abcam, USA, 1 : 1000), E-cadherin (Santa Cruz, USA, 1 : 1000), vimentin (Boster, China, 1 : 500), MMP-9 (Santa Cruz, USA, 1 : 1000), and *β*-actin (Santa Cruz, USA, 1 : 1000). After washing three times with TBS containing 0.1% Tween 20, the membranes were incubated with an HRP-conjugated goat anti-rabbit or goat anti-mouse secondary antibody (Zhongshan Golden Bridge, China, 1 : 5000) and visualized using SuperSignal West Pico Chemiluminescent Substrate (Pierce, Rockford, IL, USA).

### 2.4. Scratch Wound Assay

Migratory ability of BCPAP cells was assessed by a scratch wound assay. Cells were plated in a six-well plate and grow to confluent cell monolayers. Subsequently, three vertical scratches were made gently with sterile pipette tip across the diameter of the well and rinsed with PBS to remove debris. The wounded cell monolayer was then incubated in fresh complete medium with vehicle (DMSO) alone, 10^−8 ^M E2, 10^−6 ^M PPT, or 10^−6 ^M DPN. For each well, at least five pictures were taken microscopically at a magnification of 10x at 0 and 24 h after scratch. The percentage of nonrecovered wound area was calculated by dividing the nonrecovered area after treatments by the initial wound area at time zero.

### 2.5. Invasion Assay

Invasion assay was carried out using transwell invasion chambers (8 *μ*m pore size) in 24-well plates (Corning Life Sciences, Corning, NY, USA). Transwell was coated with Matrigel (BD Biosciences, Two Oak Park, Bedford, MA) overnight. Cells were starved for 48 hours using the starvation medium (phenol-red-free RPMI medium supplemented with 10% charcoal-dextran stripped FBS) before plating to the upper chamber. After Matrigel invasion chambers were rehydrated using the phenol-red-free and serum-free RPMI medium at 37°C, cells (2.0 × 10^4^ cells per well) in the phenol-red-free RPMI medium (100 *μ*L) with 1% charcoal-dextran stripped FBS containing vehicle (DMSO) alone, 10^−8^ M E2, 10^−6^ M PPT, or 10^−6^ M DPN were loaded onto the upper chamber, and 500 *μ*L of phenol-red-free RPMI medium with 10% charcoal-dextran stripped FBS was loaded onto the bottom chamber as a chemoattractant. After 24 hours of incubation, the noninvading cells on the upper chambers were removed with a cotton-tipped swab. Cells were fixed with 4% paraformaldehyde and stained with 1% crystal violet. The invaded cells were counted microscopically in five random fields of view at 200x magnification and expressed as the mean number of cells per field of view.

## 3. Statistical Analysis

Data were presented as mean ± SD for at least three independent replicates. Differences between groups were examined using Student's *t*-test. Differences with *P* < 0.05 were considered to be statistically significant.

## 4. Results

### 4.1. BCPAP Cells Express ER*α* and ER*β*

**Figure 1:**
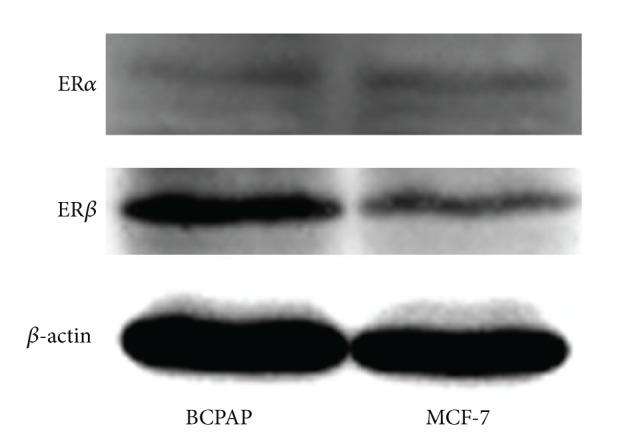
BCPAP cells express ER*α* and ER*β*. Whole cell protein was resolved by SDS-PAGE followed by western blot analysis for ER*α*, ER*β*, and *β*-actin. MCF-7 and ER-positive cells were used as positive control.

Thyroid cancer cells are not known to act as traditional estrogen-responsive tissues such as breast cancer. In order to determine the status of the ERs in BCPAP cells, western blots were performed. MCF-7, a classical ER expressing breast cancer cell line, was used as a positive control for detection of ER*α* and ER*β*. We observed that both the thyroid and breast cancer cell lines assayed expressed both ER isoforms, ER*α* and ER*β* (Figure 1). This suggests that these cells are presumably responsive to the E2-ER-mediated signaling pathway.

### 4.2. Effect of E2, PPT, and DPN on Migration of BCPAP Cells

**Figure 2:**
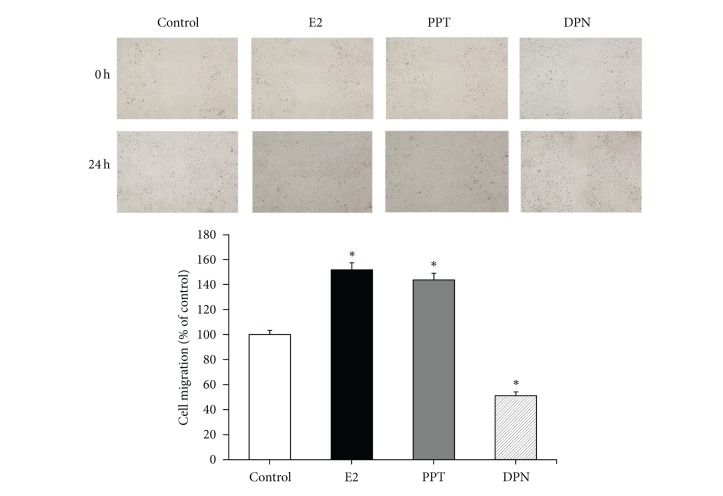
E2 enhances migration of BCPAP cells. Confluent monolayers of BCPAP cells were wounded with a uniform scratch, washed to remove cell debris, and cultured in the presence of vehicle (DMSO) alone, 10^−8 ^M E2, 10^−6 ^M PPT, or 10^−6 ^M DPN for 24 h. Images of cell cultures were captured at 0 and 24 h after scratching; representative pictures are shown in upper panel. The amount of wound repair was expressed as uncovered area at the indicated time compared with initial uncovered area of vehicle-treated control at time zero (lower panel). Values are the mean ± SD of three separate experiments (normalized to the untreated control). *P < 0.05 compared with control.

The migratory potential of BCPAP cells following E2, PPT, and DPN exposure was assayed by performing the scratch wound assay. The wounded area of BCPAP cell monolayers healed slowly in vehicle-treated cells. The closure of the wounded gap was significantly accelerated in the presence of E2 and PPT but significantly decelerated in the presence of DPN at 24 h (Figure 2). Compared with control cells, this significant increase in migration was approximately 51.6% for E2-treated cells and 43.6% for PPT-treated cells; in contrast, the significant decrease in migration was 48.9% for DPN-treated cells (normalized to 100% and *P* < 0.05) (Figure 2). This result indicates that E2 enhanced migration of BCPAP cells; ER*α* and ER*β* had opposite functions in migration of BCPAP cells.

### 4.3. Effects of E2, PPT, and DPN on Invasion of BCPAP Cells

**Figure 3:**
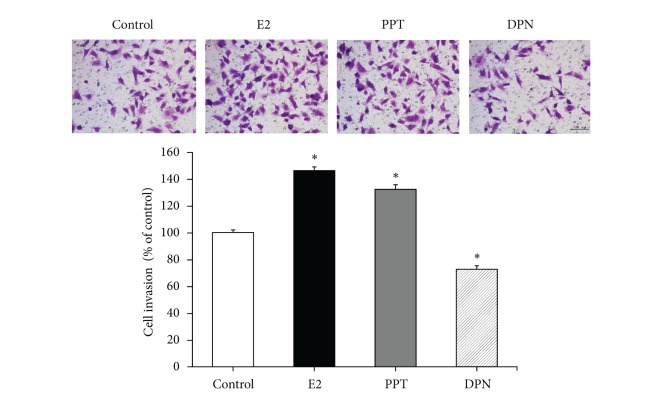
E2 enhances invasion of BCPAP cells. Cells (2.0 × 10^4^ cells per well) in the phenol-red-free RPMI medium (100 *μ*L) with 1% charcoal-dextran stripped FBS containing vehicle (DMSO) alone, 10^−8 ^M E2, 10^−6 ^M PPT, or 10^−6 ^M DPN were loaded onto the upper chamber, and 500 *μ*L of phenol-red-free RPMI medium with 10% charcoal-dextran stripped FBS was loaded onto the bottom chamber as a chemoattractant. After 24 h, the invaded cells were counted microscopically in five random fields of view at 200x magnification and expressed as the mean number of cells per field of view. Values are the mean ± SD of three separate experiments (normalized to the untreated control). *P < 0.05 compared with control.

The invasive potential of BCPAP cells following E2, PPT, and DPN exposure was assayed by the invasion assay. We observed that cell invasion increased in the presence of E2 and PPT but decreased in the presence of DPN (Figure 3). Compared with control cells, this increase in invasion was approximately 46.2% for E2-treated cells and 32.2% for PPT-treated cells; in contrast, this decrease in invasion was 27.3% for DPN-treated cells (normalized to 100% and *P* < 0.05) (Figure 3). This result indicates that E2 enhanced invasion of BCPAP cells; ER*α* and ER*β* had opposite functions in invasion of BCPAP cells.

### 4.4. E-Cadherin, Vimentin, and MMP-9 Expression following E2, PPT, and DPN Exposure

**Figure 4:**
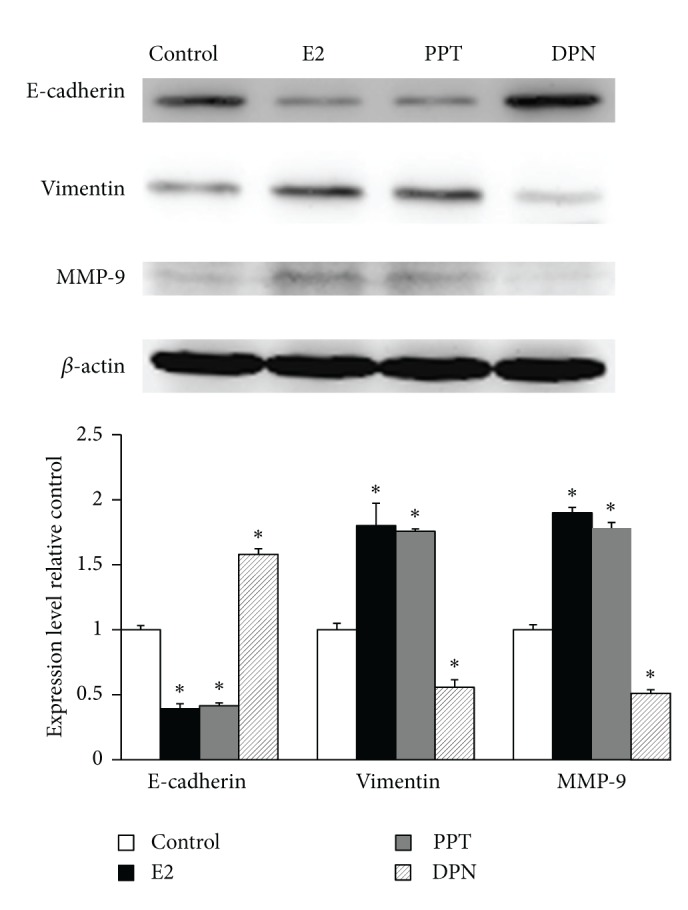
E2 induces metastasis via downregulation of E-cadherin and upregulation of vimentin and MMP-9. BCPAP cells were treated with vehicle (DMSO) alone, 10^−8 ^M E2, 10^−6 ^M PPT, or 10^−6 ^M DPN. Whole cell lysates were extracted, and E-cadherin, vimentin, and MMP-9 protein were detected by western blot analysis. *β*-actin was used as a loading control. *P < 0.05 compared with control.

To ascertain the molecular basis of E2, PPT, and DPN on modulation of metastatic potential of BCPAP cells, we analyzed expression of E-cadherin, vimentin, and MMP-9 protein by western blot analysis in BCPAP cells treated with vehicle (DMSO) alone, 10^−8 ^M E2, 10^−6 ^M PPT, or 10^−6 ^M DPN. The 24-hour treatment of BCPAP with E2 and PPT caused a significant decrease in expression of E-cadherin, but a significant increase in expression of vimentin and MMP-9. In contrast, the 24-hour treatment of BCPAP with DPN caused a significant increase in expression of E-cadherin, but a significant decrease in expression of vimentin and MMP-9 (Figure 4). These observations are suggestive of a possible role of E-cadherin, vimentin, and MMP-9 in migration and invasion of BCPAP cells regulated by E2, PPT, and DPN. 

## 5. Discussion

In the present study, we confirmed that both ER*α* and ER*β* were expressed in the PTC cell line BCPAP and E2 could induce metastatic potential of BCPAP. This is consistent with the results of previous studies that ER*α* and ER*β* were expressed in the PTC line KAT 5, NPA 87, and BCPAP, and E2 could enhance adhesion, migration, and invasion of BCPAP cells [8, 9]. Moreover, we further found that the two ER subtypes play differential roles in modulation of BCPAP cell metastasis and the related molecule expressions including E-cadherin, vimentin, and MMP-9.

Given the importance of E2 demonstrated by previous studies, understanding the mechanistic basis through which it controls thyroid carcinogenesis will have profound biological and medical implications. In fact, the proliferative effects of E2 in thyroid cancer were found to be mediated through regulation of genes involved in growth control, such as bcl-2, Bax, and c-fos [10, 18]. In contrast to the role in regulating cell proliferation, very little is known about whether and how E2 promotes thyroid cancer cell metastasis. Rajoria et al. reported that E2 could enhance adhesion, migration, and invasion of BCPAP cells through *β*-catenin and MMP-9 [8, 19]. The studies on other types of cancer have indicated that E2 may promote tumor metastasis through activation of genes linked to cell metastasis. E2 has been found to increase the metastatic potential of human epithelial ovarian cancer cell through upregulation of MMP-2 and downregulation of E-cadherin [14, 20]. E2 enhanced breast cancer cell motility and invasion via extranuclear activation of actin-binding protein ezrin [16]. E2 promoted lung cancer cell migration through downregulation of E-cadherin and *β*-catenin and upregulation of fibronectin and vimentin [17]. In this regard, our data implied that E2-induced metastatic potential of BCPAP cells was, at least in part, mediated through downregulation of E-cadherin and upregulation of vimentin and MMP-9. 

In our recent study, we found that ER*α* and ER*β* protein expression was lost in 52.8% and 1.9%, respectively, of the 106 PTC lesions (unpublished data). This implicates that ER subtypes may play the distinct roles in thyroid carcinogenesis. However, their functions and the molecular mechanisms in thyroid carcinogenesis have just begun to be unveiled. Zeng et al. reported that ER*α* promoted cell proliferation, but ER*β* induced cell apoptosis in thyroid cancer [10]. Park et al. also found that E2 triggered the metastatic behaviors exclusively through an ER*α*-dependent pathway, but ER*β* had an opposing action on ER*α* in ovarian cancer [14]. To dissect which ER subtype plays a dominant role in the prometastatic effect of E2, we treated BCPAP with the preferential ER*α* agonist PPT or with the ER*β* agonist DPN. We found that PPT enhanced, but DPN inhibited, migration and invasion of BCPAP cells through the differential regulation of E-cadherin, vimentin, and MMP-9.

The findings in this study have been limited to the typical PTC cell line—BCPAP. We would verify them in other PTC cell lines and through other methods such as ER gene knockdown technique in the future study. 

## 6. Conclusions

As a whole, our data suggest a strong link between E2 and BCPAP cell metastasis as evidenced by its effect on *in vitro* migration and invasion. We found that E2 exposure also led to downregulation of E-cadherin and upregulation of vimentin, and MMP-9. Furthermore, ER*α* and ER*β* play the distinct roles in BCPAP cell metastasis through the differential regulation of E-cadherin, vimentin and MMP-9. It carries clinical implications for selective targeting of the ERs in therapeutic and prevention strategies against thyroid cancer.
